# A Chemosensor Based on Gold Nanoparticles and Dithiothreitol (DTT) for Acrylamide Electroanalysis

**DOI:** 10.3390/nano11102610

**Published:** 2021-10-04

**Authors:** Shahenvaz Alam, Shine Augustine, Tarun Narayan, John H. T. Luong, Bansi Dhar Malhotra, Sunil K. Khare

**Affiliations:** 1 Enzyme and Microbial Biochemistry Laboratory, Department of Chemistry, Indian Institute of Technology Delhi, Hauz Khas, New Delhi 110016, India; shan45417@gmail.com; 2 Nanobioelectronic Laboratory, Department of Biotechnology, Delhi Technological University, Shahbad Daulatpur, Bawana, New Delhi 110042, India; shine2089@gmail.com (S.A.); narayantarun41@gmail.com (T.N.); bansi.malhotra@gmail.com (B.D.M.); 3 School of Chemistry, University College Cork, T12 YN60 Cork, Ireland; j.luong@ucc.ie or

**Keywords:** acrylamide, dithiothreitol, differential pulse voltammetry, impedance spectroscopy, surface plasmon resonance

## Abstract

Rapid and simple electroanalysis of acrylamide (ACR) was feasible by a gold electrode modified with gold nanoparticles (AuNPs) and dithiothreitol (DTT) with enhanced detection sensitivity and selectivity. The roughness of bare gold (Au) increased from 0.03 μm to 0.04 μm when it was decorated with AuNPs. The self-assembly between DTT and AuNPs resulted in a surface roughness of 0.09 μm. The DTT oxidation occurred at +0.92 V. The Au/AuNPs/DTT surface exhibited a surface roughness of 0.24 μm after its exposure to ACR with repeated analysis. SEM imaging illustrated the formation of a polymer layer on the Au/AuNPs/DTT surface. Surface plasmon resonance analysis confirmed the presence of AuNPs and DTT on the gold electrode and the binding of ACR to the electrode’s active surface area. The peak area obtained by differential pulse voltammetry was inversely proportional to the ACR concentrations. The limit of detection (LOD) and the limit of quantitation (LOQ) were estimated to be 3.11 × 10^−9^ M and 1 × 10^−8^ M, respectively, with wide linearity ranging from 1 × 10^−8^ M to 1 × 10^−3^ M. The estimated levels of ACR in potato chips and coffee samples by the sensor were in agreement with those of high-performance liquid chromatography.

## 1. Introduction

Acrylamide (ACR) was first identified in food products by a Swedish scientist [1], as starch-based foods were often cooked above 120 °C [2]. ACR has been marked as a Group 2A carcinogen, according to the International Agency for Research on Cancer [3]. Since its discovery in foodstuffs, the subject of the toxicity and metabolism of ACR has become a topic of debate. Based on the Maillard reaction, asparagine and starchy compounds react to form ACR at elevated temperatures [4]. The food industry needs to monitor and reduce the ACR concentrations in their products. ACR is also found in a plethora of products, particularly as an impurity in non-toxic polyacrylamide. The average ACR in processed cereal products and coffee substitutes ranges from 40 to 4000 µg/kg [5]. Cytotoxicity is linked to its consumption at elevated levels, as ACR is neurotoxic and provokes oxidative stress and apoptosis in the cells [6]. ACR forms an adduct with *N*-(2-carbamoylethyl) valine hemoglobin (Hb) [7]. ACR with rapid solubility and mobility in water has a high risk of contamination into surface and groundwater supplies. ACR and polyacrylamide have been widely used in the production of plastics, dyes, and paper, etc. Due to its toxicity, ACR has been studied using different model animals to decipher its toxic behavior. Epoxy glycidamide (GA), an oxidized metabolite of ACR, is genotoxic and forms a GA-DNA adduct inside the cells [8]. The IC (inhibition concentration) of ACR for human cell lines A549 is 4.5 mM [9]. 

Detection of ACR in foodstuffs has raised much curiosity about the presence and levels of ACR in food products. Different techniques have been employed for the detection of ACR. The extraction of ACR in fried potato peel at different temperatures was reported [10]. The determination of ACR was based on LC-MS/MS using [^13^C_3_] ACR as the labeled standard; GC/MS [11], HPLC [12], HPLC-MS/MS [13], UPLC [14], and UPLC-MS/MS [15] have also been used for the quantification of ACR. Such procedures require high costs, handling expertise, and sampling precision. 

A glassy carbon (GC) electrode was modified with single-walled carbon nanotubes for interaction with hemoglobin [16]. A nanocomposite consisting of Hb immobilized on carbonylated multi-walled carbon nanotubes/iron oxide/chitosan was used for the detection of ACR [17]. Of notice is the use of a Hb and gold nanoparticles modified nitrogen terminated boron-doped diamond (BDD) electrode [18] and Hb nanoparticles on an Au electrode for ACR analysis [19]. Anti-ACR antibodies assembled on a glassy carbon plate [20] and polyclonal antibodies specific to ACR-4-MPA on an antigen modified SnO_2_-SiC-HSNC nanocomposite were employed for the immunosensing of ACR [21]. Mainly, these biosensors were derived from bio-based recognition molecules such as Hb, DNA, and antibodies. ACR acts on these biomolecules following Michael-type nucleophilic addition reactions where the amino (NH_2_) and sulfhydryl (SH) groups present in proteins react with ACR to form a complex. The Hb-inspired biosensors showed conjugation of NH_2_ of N-terminal valine of Hb to attach to ACR [17]. Thus, Hb had mainly been utilized for the development of a biosensor for ACR detection. However, the major limitations of Hb biosensors are selectivity and reproducibility. As mentioned earlier, Hb forms adducts with ACR that are irreversible reactions. In addition, Hb showed high reactivity towards ACR analogs, such as glycidamide and epoxide of ACR, which have a higher affinity with Hb than ACR itself.

This study describes a simple chemosensor for the detection of ACR, using dithiothreitol with two end thiol groups (DTT) as a sensing molecule. Known also as Cleland’s reagent, DTT with two thiol groups is commonly used to reduce a disulfide bond in proteins. Using DTT, the sulfhydryl (SH) groups readily react with ACR to form a complex, which are exploited for the development of the chemosensor using electroanalysis. Briefly, gold nanoparticles (AuNPs) were anchored on a gold electrode by electrodeposition to improve the active sensing area. AuNPs also served as attachment sites for DTT via self-assembly to form the Au–S bond [22]. Upon absorption, DTT rapidly covers the surface of AuNPs and provides a dense monolayer even at very low concentrations [23]. D-cysteine with a thiol group reacts with ACR to form three different Michael adducts [24,25]. To our knowledge, this is the first attempt at detecting ACR using the gold electrode modified with AuNPs and DTT.

## 2. Materials and Methods

### 2.1. Chemicals and Instruments

Auric chloride (HAuCl_4_), ACR, DL-dithiothreitol (DTT), and Oasis HLB SPE cartridges were purchased from Sigma Aldrich (St. Louis, MO, USA).Monosodium phosphate, disodium phosphate, sodium chloride, potassium chloride, sodium hydroxide, potassium hexacyanoferrate (II), and (III) were purchased from Merck. Zinc acetate and glacial acetic acid were purchased from Thermo Fisher Scientific, Waltham, MA, USA. All the solvents were analytical grade with the highest purity and used without further purification. 

Cyclic voltammetry (CV), differential pulse voltammetry (DPV), chronoamperometry, and electrochemical impedance spectroscopy (EIS) were carried out using an electrochemical analyzer (PGSTAT302N, Metrohm-Autolab, Utrecht, The Netherlands). Surface plasmon resonance (SPR) studies were performed using SPR Twingle (KEI, GK Leusden, The Netherlands) combined with an electrochemical analyzer for performing interaction studies. Electrochemical and SPR data were processed using Nova software 1.6, Utrecht, The Netherlands and kinetic evaluation software, GK Leusden, The Netherlands respectively. High-performance liquid chromatography (HPLC) was performed using HPLC-Agilent 1100 (Agilent Technologies Inc., Santa Clara, CA, USA) with UV-DAD as the detector. FESEM studies were conducted and interpreted using the FESEM Model: FEI Quanta 200 F SEM and FEI Company (North Brabant, The Netherlands). The morphologies of the bare Au, Au/AuNPs, Au/AuNPs/DTT, and Au/AuNPs/DTT with ACR electrodes were also investigated. AFM studies were performed to visualize the surface modification of the modified Au electrode (model: Bruker Multimode AFM, Billerica, MA, USA). The heterogeneity of the surface was determined by the average roughness. The scan was performed on a surface area of 10 µm × 10 µm with a tip of 1 µm size. The WSxM software tool was used for image viewing and roughness analysis [26]. EDX analysis of the modified electrode was conducted using EDX (model: Hitachi High Technologies Corporation, Tokyo, Japan). Elemental analysis was calculated for the desired modification on the electrode surface and the dispersion of gold nanoparticles.

### 2.2. Cleaning and Preparation of the Working Electrode

All electrochemical measurements were conducted in an electrochemical cell of 10 mL capacity. The gold electrode (1 mm in diameter, Metrohm, Utrecht, The Netherlands) modified with AuNPs served as the working electrode, Ag/AgCl as a reference electrode (stored at 3 M KCl), and a platinum (Pt) wire as a counter electrode. The bare gold electrode was cleaned by dipping in 0.5 M NaOH, and cyclic voltammetry (CV) was cycled from −0.4 V to +1.6 V (vs. AgCl) for ten cycles at 0.05 V/s. The gold electrode was further cleaned with alumina powder (0.3 µm in diameter) and rubbed with a polishing motion against a rough surface. The electrode was then subject to sonication, followed by rinsing with ethanol and deionized water for 10 min each [27]. The electrode was then conditioned in 1 M H_2_SO_4_ with a potential from −0.3 to +1.7 V for ten cycles at 0.3 V/s.

### 2.3. Fabrication of the Working Electrode 

The clean electrode was dipped in an auric chloride solution (10 mM auric chloride in 0.5 M H_2_SO_4_). AuNPs were electrodeposited on the electrode surface by chronoamperometry at +1 V for 20 s. The Au/AuNPs electrode was dipped in a DTT solution (1 mg/mL dissolved in 0.1 M phosphate buffer, pH 7.0 with 0.9% sodium chloride) for 30 min. AuNPs were deposited on the Au electrode surface to impart better conductivity and serve as a platform for the attachment of DTT [28]. DTT with the thiol groups self-assembled to the Au surface by the Au–S covalent bond. 

### 2.4. Extraction of ACR from Food Samples and Its Quantification Using HPLC

The extraction of ACR was carried out according to the reported method [29] with modification. Potato chips purchased from the local market (2 g) were crushed, homogenized, and admixed in 50 mL deionized water. Similarly, the instant coffee powder was procured from the market for ACR extraction. All the samples were kept for 20 min to swell and incubated at 60 °C in a shaking bath for 1 h. The samples were centrifuged at 5000 rpm for 10 min. The supernatant was taken in a fresh tube, and Carrez I and Carrez II (clarifying agents for proteins) were added (0.5 mL each) into the tube, vortexed, and kept for 15 min. Precipitated food materials were centrifuged to obtain the supernatants. The supernatants were purified using Oasis HLB SPE cartridges and then used for analysis. An appropriate amount of the extracted ACR from the chip and coffee samples was added to the working solution, and the decrease in current was observed for determination. The samples were quantified using HPLC with a flow rate of 0.300 mL/min at 210 nm wavelength. Solvents were prepared using deionized water and methanol at a ratio of 7:3. The HPLC method was used to validate the results obtained by this chemosensor. 

### 2.5. Surface Plasmon Resonance Interaction Studies 

A surface plasmon resonance (SPR) analyzer was coupled with an electrochemical analyzer and used for probing the interaction between DTT with the Au/AuNPs electrode and ACR. An SPR gold disk was modified with AuNPs and DTT using a similar method, as explained in Section 2.3. The interaction between DTT with Au/AuNPs electrode was determined by observing the change in baseline after the DTT was injected on the SPR surface and allowed to interact for 30 min in PBS buffer, pH 7.0. The ACR and DTT interaction was further examined by observing the change in the baseline. 

### 2.6. Electrochemical Studies of Electrode and Detection of ACR Using the Au/AuNP-Modified Electrode

Cyclic voltammetry (CV) was conducted to have a better insight into the modification and deposition of AuNPs and DTT on a bare Au electrode. The CV was conducted where the starting potential applied was −0.5 to 1.0, with a scan rate of 50 mV/s. The CV curve was plotted by adding different concentrations of ACR in 0.1 M PBS, pH 7. EIS was conducted in the range of 0.01 kHz to 100 kHz at 10 mV in 0.1 M PBS, pH 7.0 containing 0.1 M KCl, and a mixture of K_3_[Fe (CN)_6_]/K_4_[Fe (CN_)6_]. The Nyquist plot was simulated according to the Randles equivalent circuit with double-layer capacitance (C_dl_). Using the Nyquist plot, the charge transfer resistance was calculated. The pH studies were conducted using DPV with an initial potential of −0.5 V to the end potential of +1.1 V in 0.1 M phosphate buffer with the pH ranging from pH 6.0 to 8.0. Chronoamperometry was used to determine the desorption on the electrode surface by providing a current for 2 min. Studies were conducted with the modified electrode with/without ACR. DPV was used for the determination of ACR by the AuNPs/DTT modified electrode. The calibration curve was established by plotting peak current vs. ACR concentration, and was used for the estimation of the LOD and LOQ. The ACR contents in coffee and potato chips samples were quantified using the standard calibration curve established for the modified electrode, and compared with the HPLC results.

### 2.7. Interference Study

An interference study was conducted using potential interfering compounds commonly found in food samples. Compounds such as citric acid, glycine, *L*-asparagine, sucrose, glucose, *L*-glutamate, *L*-aspartic acid, and calcium chloride were used along with ACR (analyte). All the compounds were added with a similar concentration of 1 µM. 

## 3. Results and Discussions

### 3.1. CV Analysis of Deposition of Gold Nanoparticles (AuNPs) and DTT on Bare Au Electrode 

**Figure 1:**
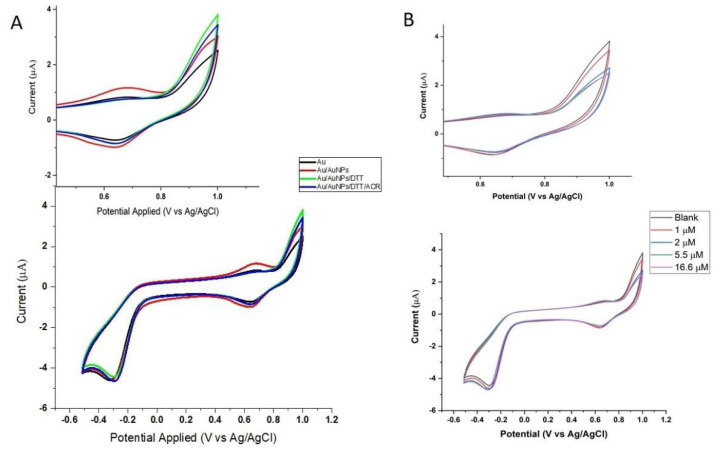
(**A**) CV analysis of bare gold, AuNP-modified Au electrode, and DTT-AuNP modified Au electrode and the addition of ACR of the AuNP-DTT modified gold electrode. (**B**) The effect of different concentrations of ACR on the modified electrode; Blank (AuNP-modified Au electrode without analyte). The observed current decreased with increasing ACR concentrations.

CV analysis (Figure 1A) was conducted by applying potential from −0.5 V to +1.1 V (vs. AgCl). The bare Au electrode exhibits the gold oxide formation and reduction (Ox/Re) region, as is well known in the literature. The oxidation peak is expected to stem from a one-electron transfer gold oxidation process (Au^0^ → Au^+^ + e^−^), which forms a neutral Au(I) complex with chloride, as PBS is used as the electrolyte. The complexation with the ligand would stabilize the Au(I) oxidation state on the gold surface (Au^+^ + Cl^−^ → AuCl_solid_) [30]. Note that Au(III) is generated at potentials above +1.3 V (vs. RHE) or ~+1.1 V (vs. AgCl) [31]. Therefore, the oxidation peak is unlikely, due to the oxidation of the gold electrode to Au(III). The oxidation peak at +0.65 V showed an increase in the peak height when AuNPs were deposited on the surface of the bare Au electrode. Similar behavior was also noted for the reduction peak, indicating the higher free concentration of Au(I) at the electrode. After the self-assembly of DTT on AuNPs (Au–S bond), the oxidation peak at +0.65 V was noticeably decreased, even lower than that of the bare gold electrode (Figure 1A). In contrast, the CV showed a noticeable increase in the current at +0.9 V (vs. AgCl) when the bare gold electrode modified by gold nanoparticles was subject to DTT. Together with the impedance measurement, as addressed later, such results evinced the formation of DTT on the gold surface. It was further confirmed that the oxidation current at +0.9 V (vs. AgCl) decreased gradually with increasing ACR concentration (Figure 1B). This observation was then exploited for the detection of ACR using the DTT-AuNP modified gold electrode.

### 3.2. Characteristics of the Bare Au Electrode

**Figure 2:**
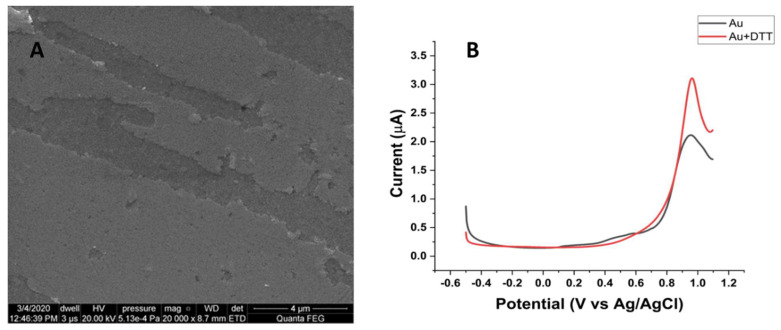
(**A**) SEM micrograph of the bare electrode illustrates the surface is least heterogeneous with an average surface roughness of 0.03 µm. (**B**) DPV of the bare gold electrode in 0.1 M phosphate buffer, pH 7.0 (black curve) with DTT adsorbed on the gold surface (red curve).

**Figure 3:**
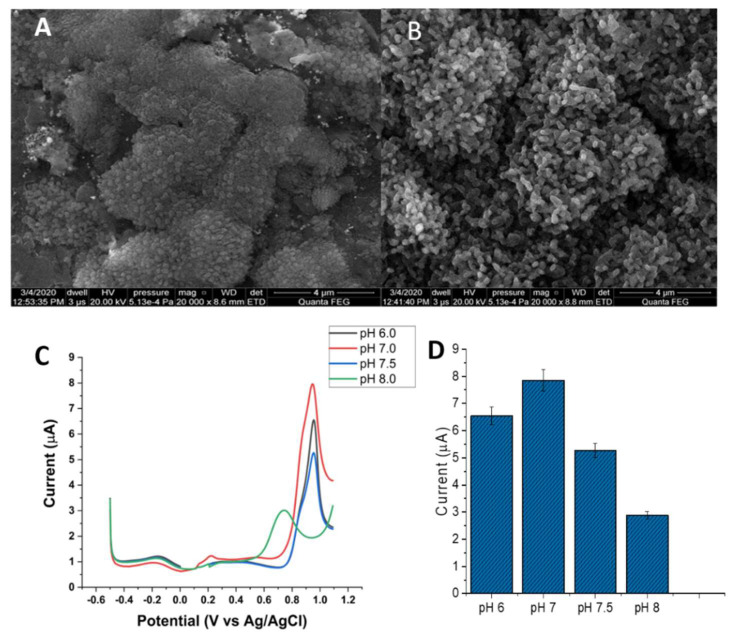
(**A**) A typical SEM micrograph of bare gold electrode decorated by gold nanoparticles. (**B**) An SEM micrograph depicts the self-assembly of DTT to AuNPs of the Au/AuNPs electrode. (**C**) DPV of the Au/AuNPs/DTT electrode in 0.1 M phosphate buffer at 4 different pHs. (**D**) Current intensity of the electrode at different pHs, 6.0 to 8.0.

As expected, bare Au was the least heterogeneous, as illustrated by the SEM micrographs, (Figure 2A and Figure 3A). and the WSxM tool from its AFM micrograph estimated an average surface roughness of 0.03 µm.

DPV, with an initial potential of −0.5 V to the end potential of +1.1 V, was used with a step potential of 0.005 V at 0.01 V/s. DPV of the bare electrode exhibited one single peak at +0.92 V, which is well-known as the oxygen evolution peak [32]. At this potential, the hydroxyl (OH•) radical formed during water electrolysis is highly reactive to dimerize into hydrogen peroxide (H_2_O_2_), which is further oxidized into the O_2_ molecule. The experiment was then conducted to investigate the DPV behavior of bare Au with DTT simply adsorbed on its electrode surface. The bare Au electrode with adsorbed DTT displayed a higher and sharper oxidation peak at +0.92 V (Figure 2B), indicating the oxidation of DTT.

**Scheme 1:**
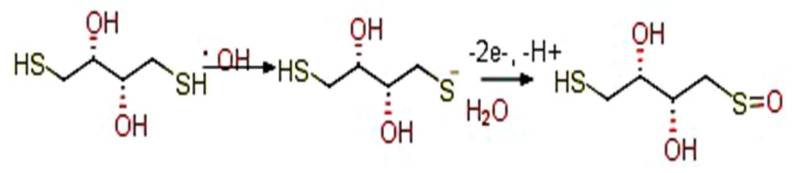
The oxidation of DTT_red_ to DTT_ox_ involves two electrons and one H^+^. Without the radical, the SH group is not active, and DTT remains in the reduced form.

DTT has little tendency to be oxidized directly by air, compared to other thiol compounds. It has the advantage to serve as a protective reagent with two thiol groups and redox potentials of −0.33 V at pH 7.0 and −0.366 V at pH 8.1 [33]. With DTT adsorbed on the bare gold, the thiol group with the lower pKa = 8.3–9.1 is deprotonated by the OH• radical [34] and further oxidized, as follows (Scheme 1).

### 3.3. Characteristics of the Au Electrode Modified with Gold Nanoparticles (AuNPs)

Among the various procedures [35], the electrodeposition of AuNPs on bare gold is the simplest procedure with controllable particle sizes and densities of AuNPs on bare gold [36] shown in Figure 3A.After incubation with DTT, the surface roughness of the Au/AuNPs/DTT was determined to be 0.09 µm as one thiol group (–SH) of DTT was self-assembled to AuNPs to form a covalent Au–S bond. Since thiol is known to bind gold to form an Au–S bond with a high affinity, the binding event does not associate any reaction. The formation of an Au–S covalent bond involves the dissociation of the S–H bond, followed by the formation of the AuS covalent bond [37]. The loss of hydrogen could take several minutes [38], and the resulting Au–S bond is the weakest among the covalent bonds; Si–O > Si–C > C–N > C–C > C–O > Au–S and >Au–Au [39]. The formation of the Au–S bond can vary from seconds to minutes and up to hours and days, depending on the type of thiol molecules and their concentration [40]. The attachment of –SH groups to AuNPs would not affect the binding force of Au–Au bonds, whereas the Au–S bond is sufficiently stable, enabling the fabrication of surface-assembled monolayers (SAMs) for diversified applications [39]. The Au–S bond is weak, as mentioned earlier, as the rupture force of an Au–S bond is only 1.2 nM [41] to 1.5 nM [42]. In this study, the AuNPs/Au electrode was incubated with DTT at pH 7.0 with an optimal time of 30 min. The resulting electrode was subject to several cycles of cyclic voltammetry until a stable background was attained (figure not shown). A distinct feature was noticed at the surface, as shown in Figure 3B. In brief, the adsorption of thiols onto the gold surface started with physisorption, as the large amount of DTT acted as a reduction reagent, and the oxidized gold surface was obtained by electrochemical oxidation. A chemisorption step then takes place with the breaking of the S–H bond, resulting in the formation of an Au–S bond, the deprotonation of thiols, and the formation of thiyl radicals [43]. The oxidized gold surface was then reduced during the formation of Au–S covalent bonds for the next cycle of DTT attachment. A stable baseline of CV attested to the formation of a monolayer of DTT on the Au electrode modified with AuNPs. It should be noted that an alkaline environment favors this dissociation, whereas an acidic environment inhibits the dissociation of S–H bonds. After the formation of an initial Au–S covalent bond, multiple bonding scenarios exist for other thiols, depending on the location of the H atom, owing to the possibility of forming weaker coordinate bonds between the protonated SH groups and gold surfaces [44].

The DPV of the Au/AuNPs/DTT also exhibited one large oxidation peak at +0.92 V, resulting from oxygen evolution and oxidation of DTT at this potential (Figure 3C). The DTT oxidation peak should be pH-dependent as its oxidation involves one H^+^ (Scheme 1). The potential peak shifted to more negative values with the increasing pH, and a drastic decrease in the peak intensity was noted at pH 8 (Figure 3C). Such a result was in agreement with the oxidation of DTT by a glassy carbon electrode [45]. Moreover, DTT is more robust as compared to Hb and antibodies against ACR, two biorecognition molecules for the detection of ACR [16]. Figure 3D depicts the bar chart of the peak current of the Au/AuNPs/DTT electrode at the different pH (6.0 to 8.0).

The EIS spectra obtained for bare Au, Au/AuNPs modified, and Au/AuNPs/DTT were modeled as a Randles electrical equivalent circuit. The values of Rct, or the charge transfer resistance of the three electrodes, were obtained as follows: bare Au (90.4 Ω), Au/AuNPs (31.8 Ω), and Au/AuNPs/DTT (151 Ω) (Figure S2).

Such Rct values affirmed the formation of AuNPs and DTT on the gold surface. Elemental weightage was estimated using EDX, where the deposition of DTT and ACR on the surface decreased the Au elemental weightage. AFM micrographs of bare Au, Au/AuNPs, Au/AuNPs/DTT, and Au/AuNPs/DTT exposed to ACR are shown in Figure S1. This signified the deposition of the elements on the surface of the electrode, as observed from the change in the weightage of the gold. Bare Au had a gold weightage of 85.59% (Figure S5a, while the deposition of AuNPs increased the weight % to 86.4 (Figure S5b). The deposition of DTT and ACR again slightly decreased the weight % to 85.4 and 78.67, respectively, shown in Figure S5c,d. The presence of ACR significantly decreased the weight percentage as compared to DTT.

### 3.4. Electrochemistry of the Au/AuNPs/DTT in the Presence of ACR 

ACR is considered as a high electron-deficient alkene that reacts with a free thiol group at neutral or when slightly alkaline pH [46]. The ACR moiety in N^ε^-acryloyl-lysine readily reacts with β-mercaptoethanol and DTT [47]. Thus, the Au/AuNPs/DTT modified electrode could serve as a simple chemosensor for ACR. 

**Figure 4:**
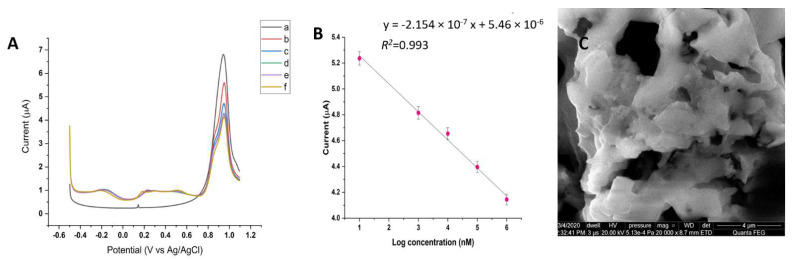
(**A**) DPV of the chemosensor in the presence of ACR. The ACR concentration (a–f) was used as follows: (a) Control (b) 1 × 10^−8^ M, (c) 1 × 10^−6^ M, (d) 1 × 10^−5^ M, (e) 1 × 10^−4^ M, (f) 1 × 10^−3^ M; (**B**) The calibration curve of standard ACR with *R^2^* = 0.993. (**C**) A representative SEM micrograph of the chemosensor surface after its exposure to ACR with an estimated surface roughness of 0.24 µm.

A series of experiments was performed to assess the DPV responses of the chemosensor in the presence of ACR. As the amount of ACR increased, the peak current decreased. Two new peaks emerged at +0.2 V and +0.6 V (Figure 4A). The peak current at +0.9 V was inversely proportional to the ACR concentration with dynamic linearity ranging from 1 × 10^−8^ M to 1 × 10^−3^ M (Figure 4B). Although the lowest concentration of ACR detected was 1 × 10^−8^ M, this chemosensor holds the potential to detect even lower than 10 nM. The slope of the linear curve was estimated to be −2.154 × 10^−7^ µA/log [C] with a correlation coefficient (*R*^2^) of 0.993. The limit of detection (LOD) was determined using the Equation (1):

LOD = 3SD × (2.303) X/The slope of the semi-log plot(1)

where ‘SD’ is the standard deviation of the blank, and ‘X’ is the lowest concentration of analyte detected, and is equivalent to the limit of quantification (LOQ). The LOD was calculated to be 3.11 × 10^−9^ M. The surface roughness of the chemosensor increased from 0.09 to 0.24 µm after its exposure to repeated analysis of ACR. SEM imaging illustrated the occurence of a similar-looking network of polymers on the electrode surface (Figure 4C). Additionally, chronoamperometry adsorption study was conducted in the absence and presence of ACR. The diffusion coefficient was calculated using Cottrell equation (Figure S3).

**Scheme 2:**
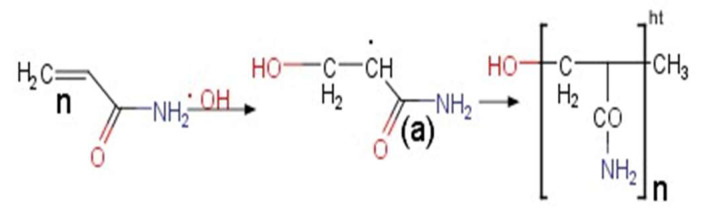
Polymerization of ACR by the hydroxyl radical.

The hydroxyl radical generated from water electrolysis, as discussed earlier, was a highly chemical-reactive species that provoked the polymerization of ACR. TiO_2_ nanoparticles under ultraviolet irradiation provided hydroxyl radicals for the polymerization of ACR [48]. Similar to chemical polymerization, ACR monomers were converted into free radicals that could proceed to react with inactivated ACR monomers (Scheme 2).

**Scheme 3:**
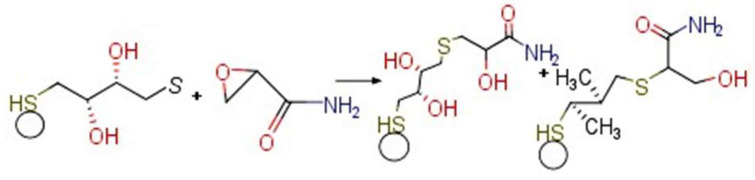
Epoxidation of ACR to GA, which is capable of conjugation with DTT bound on AuNPs. The presence of the -OH group from the ACR moiety indicates these two isomers can be electroactive.

In this context, ACR competed with DTT for the pool of hydroxy radicals, resulting in a decrease in the oxidation peak of DTT with increasing ACR concentration. The formation of the ACR polymer alone, however, could not explain the evolution of two emerging peaks in the DPV (Figure 4A). ACR must be subject to other reactions on the electrode surface under the applied potentials. The epoxidation of ACR to GA is catalyzed by the enzyme CYP2, a member of the cytochrome P450 family [49]. GA reacts with the thiol group of small organic molecules such as cysteine, glutathione, etc. [49,50]. The electrophilic double bond of ACR can participate in nucleophilic reactions with active hydrogen-bearing functional groups such as the SH of cysteine, homocysteine, and glutathione [51]. Therefore, ACR is also commonly used to selectively modify SH groups in structural and functional proteins [51]. As GA is catalyzed by the cytochrome P450 2E1 from ACR, it has been suggested that the epoxide may only be present in vivo [52,53]. Therefore, it remains to be investigated whether GA can be oxidized from DTT-bound on AuNPs, a subject of future endeavors. However, both ACR and DTT were confined on AuNPs with intimate contact, GA formed for the epoxidation of ACR should be able to conjugate with DTT bound on AuNPs to form two different isomers (Scheme 3).

ACR (–NH_2_) can be oxidized (–NH^+^) and grafted onto a GC electrode surface, but not on a Pt or Au electrode surface [54]. The cyclic voltammetric deposition is performed using 1 mM ACR solution in acetonitrile containing 100 mM NBu_4_BF_4_, an organic salt with the potential ranging from 0.0 mV to +2600 mV. Therefore, this scenario is unlikely to apply to the setup and electrochemical conditions used in this study. Of importance is the reaction between the sulfhydryl group of *d*-cysteine and the β-site double bond of ACR to form 2-amino-3-(3-amino-3-oxo-propyl)sulfanyl-propanoic acid [25]. The reaction is optimal at pH 6.5 and has a reaction time of 50 min at 90 °C. Both the reaction time and temperature are not attractive for electroanalysis as the experiment is carried out at room temperature (25 °C) in a few minutes. Nevertheless, this possibility will not be ruled out entirely as water lysis during oxygen evolution can be represented as 2H_2_O → O_2_ (gas) + 4H^+^ + 4e^−^. ACR on the Au/AuNPs/DTT surface could be oxidized and then conjugated with DTT bound on AuNPs. However, this product is not electrochemically active to account for the two emerging peaks in the DPV as shown in Figure 4A. FT-IR of the working electrode had given an insight into the Au/AuNPs/DTT in the presence of ACR. During the stepwise modification and deposition of AuNPs on the bare Au electrode, functional attributes were reflected in the FT-IR spectra. Due to the self-assembly of DTT on Au/AuNPs, a new peak emerged at 1288.26 cm^−1^ that was possibly due to S=O. Further, in the presence of ACR, prominent peaks indicated the presence of alkene (=CH_2_) at 1281.75 cm^−1^ and 1436.15 cm^−1^ (Figure S4). 

### 3.5. Interference Study 

**Figure 5:**
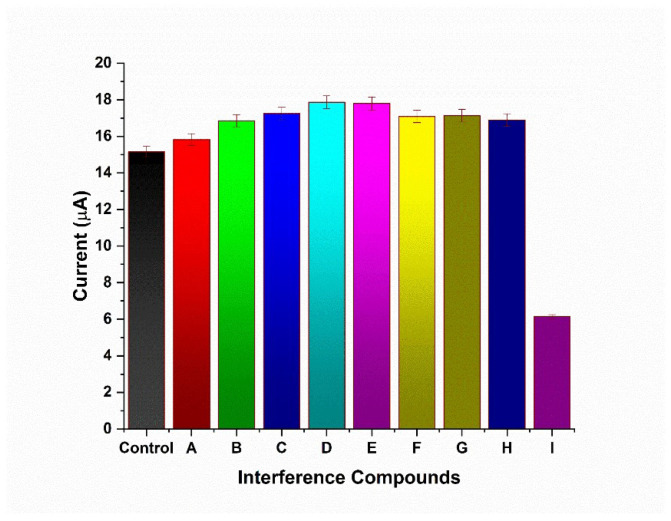
Interference study showing addition of compounds where control was chemosensor electrode without addition of compounds. (A) Citric acid; (B) A + Glycine; (C) B + L-asparagine; (D) C + Sucrose; (E) D + Glucose; (F) E + L-glutamate; (G) F + L-aspartic acid; (H) G + Calcium chloride; (I) H + ACR (analyte). All the additives were added with 1 µM concentration.

The interference study was conducted in the presence of several organic compounds, mainly found in the food samples. Compounds such as amino acids, starch, and analogous compounds with structures comparable to ACR were studied. Samples were added sequentially and ACR was added in the end. The obtained results revealed that the addition of interfering compounds had no significant effect on the current potential. As ACR was added to the buffer, the current reduced drastically by 60% from control. Figure 5 shows the interfering compounds and their plausible interference. Thus, this study indicated the high selectivity of the chemosensor toward ACR detection.

### 3.6. Surface Plasmon Resonance (SPR) Analysis 

**Figure 6:**
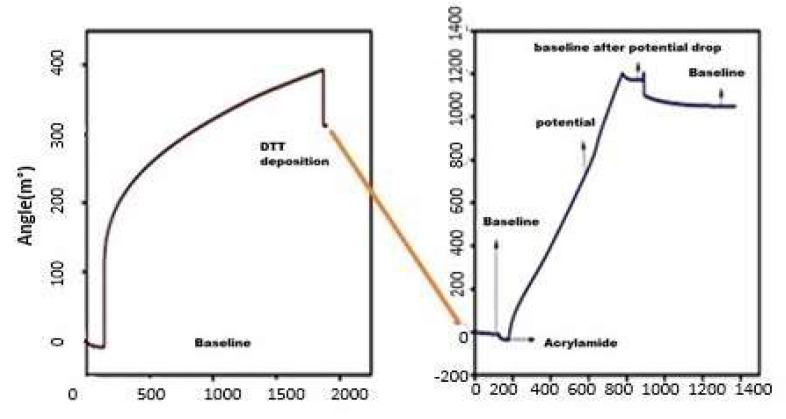
The surface plasmon resonance for interaction studies of DTT with Au electrode and with ACR. (Left) DTT showed strong bonding and conjugation with Au/AuNPs electrode. On providing potential at 0.9 V to the system, ACR also showed good interaction with DTT self-assembled to AuNPs and its plausible polymerization.

SPR was combined with an electrochemical analyzer for observing real-time changes on the surface of the electrode with each step of modification (Figure 6). Initially, AuNPs were deposited onto the Au disk electrode using chronoamperometry (1 V, 20 s). The initial baseline of the Au/AuNPs disk electrode was obtained with PBS buffer, and further DTT solution (1 mg/mL) was injected and allowed to interact for 1700 s. The surface was subsequently washed using PBS buffer until the baseline was obtained. An SPR signal jump of 311 m° from the initial baseline was observed with an increase in surface density to 2.54 ng/mm^2^, as determined from Equation (2).

Surface density = ΔResponse (m°)/conversion factor [m°·(mm^2^/ng)] (2)

The DTT-modified AuNPs/Au electrode surface was introduced with ACR (1 µM), at a potential of +0.9 V, which increased the SPR signal. After the potential drop, the baseline stabilized at 1173 m°, indicating plausible polymerization of ACR and its interaction with DTT. This was followed by washing to remove any unbound ACR molecules, leading to a decrease in the SPR signal to 1046 m°. The surface density calculated after the deposition was 8.57 ng/mm^2^. It should be noted that without the applied potential, the addition of ACR provoked no SPR response. 

### 3.7. Sensing of ACR from Food Samples

Coffee powder and potato chips were subject to extraction, and the sample with expected ACR was stored at 4 °C until use. Different amounts of samples at 10, 20, 30, and 40 µL were added to the electrolyte buffer, and the peak height was measured and calculated. As the amount of the sample increased, the peak current decreased proportionally, indicating the presence of ACR. The estimation of acrylamide concentration using HPLC is based on via a standard calibration curve of acrylamide ranging from 5–100 µg/mL (Figures S7 and S10). The water extracted samples of acrylamide from the food samples, which were subjected to the Oasis HLB cartridge and purified to remove proteins. ACR was estimated at 210 nm wavelength by the UV-Diode detector (Figures S8 and S9). The estimated concentration of ACR was 3.9 mg/kg (3.89 ppm) from the potato chips sample by the sensor, in agreement with the HPLC value of 3.54 mg/kg (3.54 ppm). Similarly, for coffee samples, the estimated concentration of ACR was 1.94 mg/kg (1.94 ppm). In comparison, ACR was estimated with an HPLC value of 1.81 mg/kg (1.81 ppm) for coffee samples (Figure S5c,d. The Australian market stipulates the maximum level of ACR in cosmetics as 5 ppm [55]. For the recovery of ACR samples from potato chips samples, the amounts of 10 nM and 15 nM were added. For coffee samples, two different concentrations of 25 nM and 50 nM were added and recoveries were determined. With a simple setup and a very low detection limit, our chemosensing approach for ACR was favourable when compared with different sensing techniques reported in the literature (Table 1).

Table 2 shows the recoveries of the spiked ACR samples. The DPV peak current of the known concentration of ACR was added into the chip and coffee samples, and the recoveries were calculated (Figure S11). 

Pertinent experimental data illustrated less interference of food constituents such as amino acids, starch, and other organic compounds to the developed sensor. Food prepared from sugars and amino acids by frying, roasting, and baking always contains some levels of ACR, ranging from <10 ppb to 8.44 ppm for sweet potato chips with sea salt crinkle-cut [40]. The ACR molecule can be readily hydrolyzed to NH4^+^, which can be detected by a selective electrode [60]. However, this approach has a strong cross-reaction to compounds containing NH_2_ groups, such as amino acids, proteins, formamide, acetamide [61], etc. The use of immunoassays deserves a brief comment here because it is a mature technology. As a small compound, ACR lacks antigenic determinants and immunogenicity. ACR must be cross-linked with a carrier protein, e.g., bovine serum albumin and ovalbumin with immune response. Polyclonal antibodies can also be raised from a hapten derived from ACR with 3-mercaptobenzoic acid [62], 4-mercaptophenylacetic-acid [63], and mercaptobenzoic acid [64], etc. Such polyclonal antibodies become specific for the haptens but not for ACR, per se. As mentioned earlier [16], Hb has been attempted for the detection of ACR. In brief, the electrical activity of Hb is based on the reversibility of Hb-Fe^3+^ to Hb-Fe^2+^. The valine -NH_2_ in Hb can form a complex with ACR to decrease the amount of Hb-Fe^2+^, i.e., the electron transfer on the electrode surface. As the heme group is embedded inside a polypeptide chain, the electron transfer is not rapid, resulting in sluggish responses. Of interest is the preparation of Hb nanoparticles (HbNPs), which are covalently immobilized on a polycrystalline Au electrode [19]. This biosensor has a response time of 2 s and a LOD of 0.1 nM for ACR. A screen-printed Au electrode is modified by a double-stranded DNA/Hb for the detection of ACR [56]. Based on square wave voltammetry, the sensor exhibits a linear working range of ACR from 2 × 10^−6^ to 5 × 10^−2^ M and a LOD of 1.58 × 10^−7^ M. A boron-doped diamond electrode can also be modified by platinum nanoparticles and Hb for the detection of ACR, with ACR concentrations ranging from 0.01–1.00 nM [65].

## 4. Conclusions

A gold electrode was modified with gold nanoparticles, and DTT was applicable for the fast and accurate determination of ACR in potato chips. It should be noted that the sensing approach did not involve the use of expensive chemicals or expensive biomolecules. Majorly, ACR sensors developed so far consisted of biomolecules such as hemoglobin and DNA. These biomolecules represent a binding with ACR with a complex cascade of reactions. This developed chemosensor with a simple chemical conjugation has been proven as an easy tool for the detection of ACR. In comparison with Hb-based biosensors, the electrode could be used multiple times and stored at room temperature without any changes in the performance of the electrode. For repeated analyses, the electrode needs to be reconditioned electrochemically. LOD and LOQ were calculated as 3.11 × 10^−9^ M and 1 × 10^−8^ M, respectively, compared with other developed biosensors. The levels of ACR concentrations in potato chips and coffee powders obtained by the fabricated chemosensor were in agreement with those of HPLC. ACR is dangerous at concentrations of 0.06 mg/L (0.844 µM = 844 nM) [66], significantly higher than the LOD of the developed chemosensor. Thus, ACR contamination into the environment is a significant threat, and this compound can be easily monitored by simple chemosensing. This chemosensing approach opens the way for the detection of other amide-containing herbicides, such as dimethenamid (a widely used herbicide) and napropamide (N,N-diethyl-2-(naphthalen-1-yloxy) propenamide, a monocarboxylic acid amide) in the agricultural fields and the environment.

## Figures and Tables

**Table 1:** A Comparison of Different Electrode Systems for Detection of ACR.

Scheme	Electrode Composition	LOD	Sample Type	Reference
1	Double-stranded DNA (dsDNA)/(Hb) modified screen-printed gold electrode	0.15 µM	Potato fries	[56]
2	Electrode with modified cobalt-phthalocyanine with GSH enzyme coupling	50 nM	Bread and potato chips	[57]
3	Carboxylic-modified single-walled carbon nanotube screen-printed electrodes	30 nM	Fried potatoes	[58]
4	Gold nanoparticles (AuNPs) and FAM-labelled double-stranded DNA (FAM-dsDNA)	10 nM	Tap water and potato chips	[29]
5	Single-stranded DNA on a gold electrode	8.1 nM	Tap water and potato chips	[59]
6	Gold electrode/Gold nanoparticles/DTT	3.11 nM	Potato chips and coffee	Present study

**Table 2:** Recoveries of ACR in Food Samples.

Food Sample	Concentration Added (nM)	Concentration Detected (nM)	Recovery (%)
Chips	10	9.55	95.5
Chips	15	12.11	81
Coffee	25	28.54	114.17
Coffee	50	46.77	93.55

## Data Availability

Data is contained within the article or Supplementary Material.

## Supplementary Materials

The following are available online at https://www.mdpi.com/article/10.3390/nano11102610/s1, Figure S1: AFM micrograph showing depo-sition of AuNPs, DTT and ACR on bare Au electrode; Figure S2: Nyquist plots for different modi-fied electrodes: bare Au, Au/AuNPs, and Au/AuNPs/DTT. Inset-Randles equivalent circuit shows a double-layer capacitance that is used to plot the simulation; Figure S3: Chronoamperometry studies of the developed sensor when the current was plotted vs. time, in presence and absence of ACR; Figure S4: FT-IR of sequential modification of bare Au with AuNPs, DTT, and ACR; Figure S5: (a) EDX of bare Au was done and showed the weight% of gold up to 85.59%; (b) weight% up to 86.48% of gold as the deposition of AuNPs on the Au electrode; (c) As DTT was added onto the Au/AuNPs electrode, there was a slight change on the surface. Gold weight % was estimated to be 85.64%; (d) The deposition of ACR on the Au/AuNPs/DTT fabricated electrode results in a decrease in the weight% of gold up to 78.67%; Figure S6a DPV curve when chips sample with an unknown concentration was added in the electrolyte solution; Figure S6b DPV curve of coffee samples with an unknown concentration differing volume of samples was added in the electrolyte solution; Figure S7. A representative HPLC chromatogram of standard ACR with a retention time of 6.3 min; Figure S8. A representa-tive HPLC chromatogram of the extraction of ACR (Peak Area-93844) from the chip sample with a retention time of 6.0 min; Figure S9. HPLC chromatogram of the coffee sample where ACR was observed at a retention time of 6.35 min; Figure S10. The HPLC calibration curve of the standard ACR with different concentrations (5, 10, 20, 50, and 100 µg/mL); Figure S11a The DPV current peak of ACR was added to chips samples. (A) Peak without the addition of analyte; (B) Addition of 10 nM ACR and (C) 15 nM ACR; Figure S11b The DPV current peak of ACR at the addition of ACR with 25 nm and 50 nm concentration.
