# Supplementary material for: A Chemosensor Based on Gold Nanoparticles and Dithiothreitol (DTT) for Acrylamide Electroanalysis
Source: Nanomaterials (Basel). 2021 Oct 4;11(10):2610. doi: 10.3390/nano11102610 (PMC8540553; doi:10.3390/nano11102610)
Supplement: Supplementary file 1 [file nanomaterials-11-02610-s001.zip › nanomaterials-1369709-supplementary.pdf]

nanomaterials

Supplementary Materials

A Chemosensor Based on Gold Nanoparticles and Dithiothreitol (DTT) for Acrylamide Electroanalysis

Shahenvaz Alam [1], Shine Augustine [2], Tarun Narayan [2], John H.T. Luong [3], Bansi Dhar Malhotra [2] and Sunil K. Khare [1,*]

1 Enzyme and Microbial Biochemistry Laboratory, Department of Chemistry, Indian Institute of Technology Delhi, Hauz Khas, New Delhi-110016, India; shan45417@gmail.com (S.A); skkhare@chemistry.iitd.ac.in (S.K.K.)
2 Nanobioelectronic Laboratory, Department of Biotechnology, Delhi Technological University, Shahbad Daulatpur, Bawana, New Delhi-110042, India; shine2089@gmail.com (S.A.); narayantarun41@gmail.com (T.N.); bansi.malhotra@gmail.com (B.D.M.)
3 School of Chemistry, University College Cork, Cork T12 YN60, Ireland; j.luong@ucc.ie or luongprof@gmail.com
* Correspondence: skkhare@chemistry.iitd.ac.in

1. AFM

AFM was performed to study the heterogeneity and properties of the electrode surface. As expected, bare Au was least heterogeneous, with an average surface roughness of 0.03 μm as estimated by the WSxM tool. After the deposition of AuNPs, roughness increased to 0.04 μm, indicating the formation of 10 nm nanoparticles in diameter on the gold surface. The roughness, however, increased to 0.09 μm for Au/AuNPs/DTT and 0.24 μm for Au/AuNPs/DTT with acrylamide (ACR).

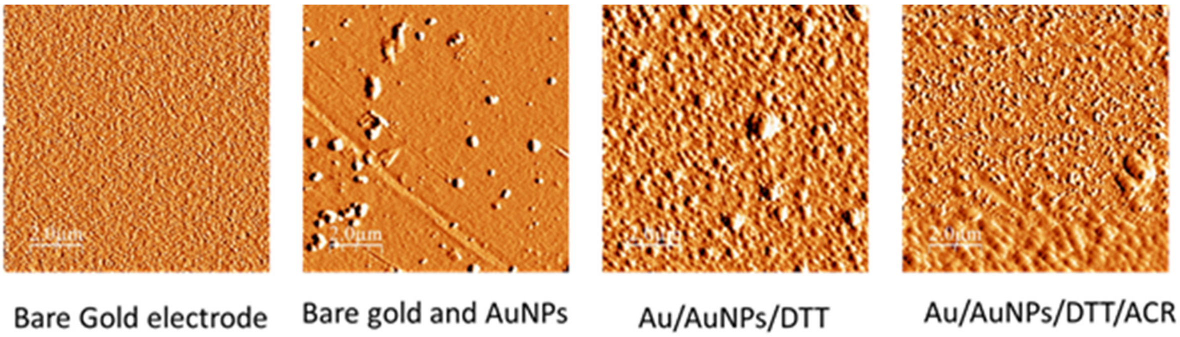

Figure S1. AFM micrograph showing deposition of AuNPs, DTT and ACR on bare Au electrode.

2. Impedance Spectroscopy

The EIS spectra modeling for bare Au, Au/AuNPs modified, and Au/AuNPs/DTT was done with the Randles electrical equivalent circuit. The arrangement consists of solutions resistance, Rs (resistance of electrolyte solution) connected to double layer capacitance in series, and double layer capacitance is connected parallel to R_{ct} (charge transfer resistance) and Warburg impedance (Z_w).

R_{ct} is the charge transfer resistance of the electrode surface, and *n* is the number of charges transferred electrons per molecule of redox probe (n = 1) for the $[Fe(CN)6]^{3-/4-}$ probes. R_{ct} was calculated according to the Nyquist plot with simulation using Nova software 1.6. As the modification and deposition were done on the gold electrode's surface, the charge transfer resistance increased due to limited electron transfer in the electrolyte solution and at the surface of the electrode. The semi-circular obtained after DTT's addition showed electrolytic resistance [1].

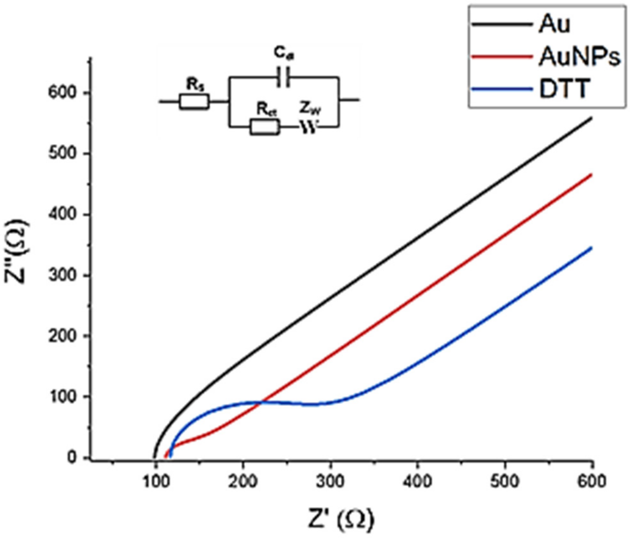

Figure S2. Nyquist plots for different modified electrodes: bare Au, Au/AuNPs, and Au/AuNPs/DTT.Inset-Randles equivalent circuit shows a double-layer capacitance that is used to plot the simulation.

3. Chronoamperometry Adsorption Studies

Chronoamperometry was performed using the modified electrode and scan for 120 s where the current was plotted vs. time. As the analyte was added, the current magnitude decreased, indicating the analyte diffusion from the bulk electrolyte solution to the electrode surface [2]. Figure S3 shows the adsorption study conducted on the fabricated electrode with/without ACR. The diffusion coefficient was calculated according to the Cottrell equation described as Equation (1): [3]

$$i_d = nFAD^{1/2}C^{*}\pi^{-1/2}t^{-1/2} \quad (1)$$

where i_d is the diffusion current, n is the number of electrons transferred in the electrochemical reaction step, F is the Faraday constant, A is the active electrode area, D is the diffusion coefficient of analyte in the electrolyte, C^* is the initial bulk concentration of the analyte in electrolyte and t is the time. This equation is valid for the electrode in an undisturbed electrolyte solution. The diffusion coefficient was calculated by using the slope of the graph plotted between i_d and $t^{1/2}$. The diffusion coefficient was found to be 2.065×10^{-9} cm^2 s^{-1}. In reported literature, ACR polymers such as 2-hydroxy-5-bromobenzaldehyde polyacrylamide (PABSB) had a diffusion coefficient of 3.129×10^{-8} cm^2 s^{-1} comparable to the obtained diffusion coefficient [4].

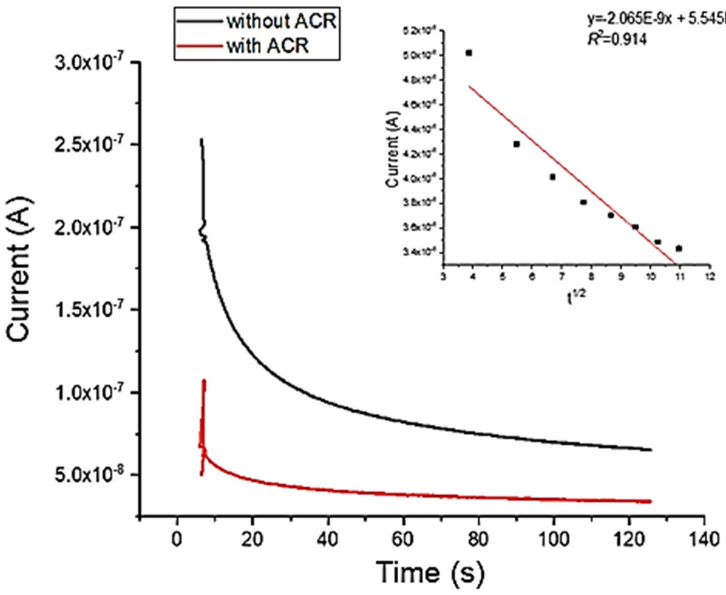

Figure S3. Chronoamperometry studies of the developed sensor when the current was plotted vs. time, in the presence and absence of ACR.

4. FT-IR Spectroscopy for the Modification of Chemosensor

FT-IR was performed to determine the modifications of the chemosensor. Sequential modifications of AuNPs and DTT on the bare Au electrode showed changes in the functional group. Due to the self-assembly of DTT on Au/AuNPs, a new peak emerged at 1288.26 cm^{-1}, which was possibly due to S=O. In the presence of ACR, peaks determining alkene (=CH_2) at 1436.15 cm^{-1}.

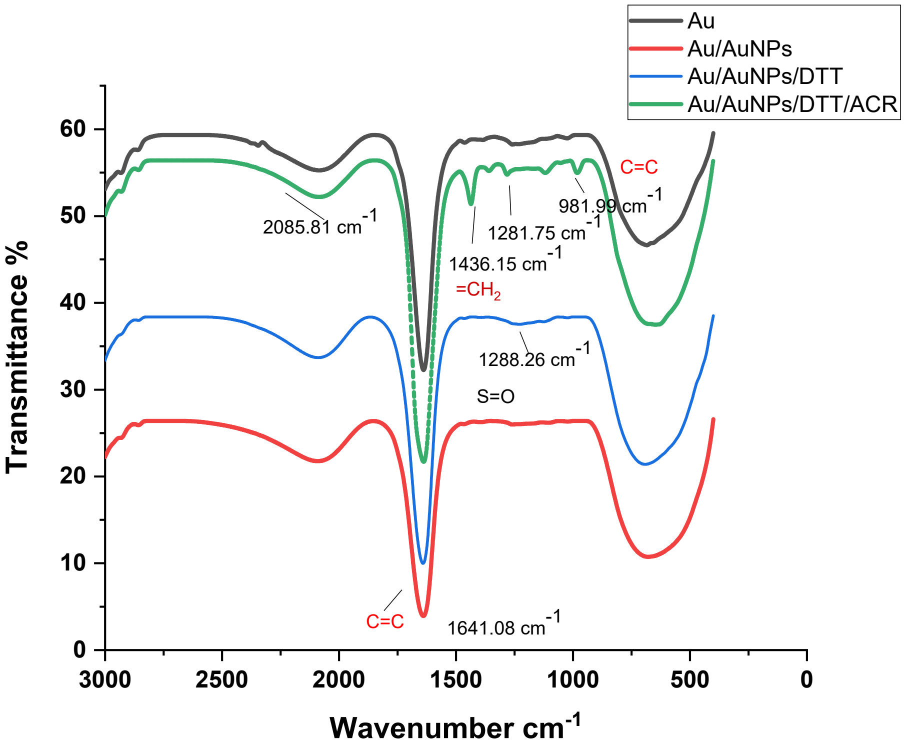

Figure S4. FT-IR of sequential modification of bare Au with AuNPs, DTT, and ACR.

5. EDX for Elemental Analysis of the Modified Electrode

Elemental weightage was calculated for every modification on the electrode surface, and dispersion of gold with modification was estimated. On modification, gold weightage was found to be 85.59% (Figure S5a). AuNPs deposition increased the weightage up to 86.48%, shown in Figure S5b. Adding DTT and ACR decreased the weightage to 85.64% and 78.6%, respectively (Figure S5c,d).

(a)

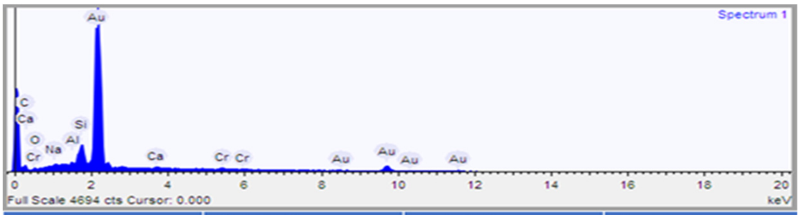

Element	Weight %	Weight % σ	Atomic %
Carbon	7.061	0.559	44.039
Oxygen	2.616	0.435	12.249
Sodium	0.497	0.109	1.619
Aluminum	0.392	0.076	1.088
Silicon	2.158	0.100	5.755
Calcium	0.632	0.109	1.181
Chromium	1.053	0.160	1.517
Gold	85.591	0.685	32.552

(b)

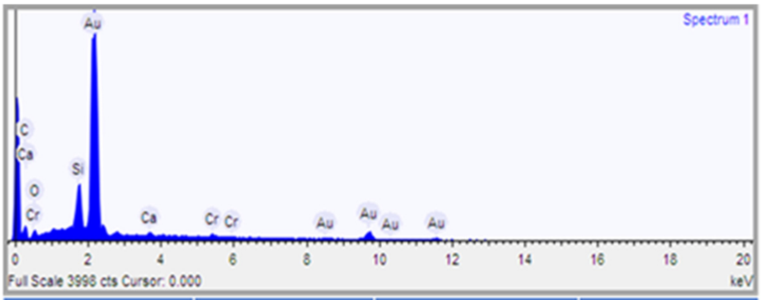

Element	Weight %	Weight % σ	Atomic %
Carbon	7.721	0.572	48.258
Oxygen	2.271	0.458	10.654
Silicon	2.477	0.104	6.620
Chromium	1.043	0.164	1.506
Gold	86.488	0.699	32.963

(c)

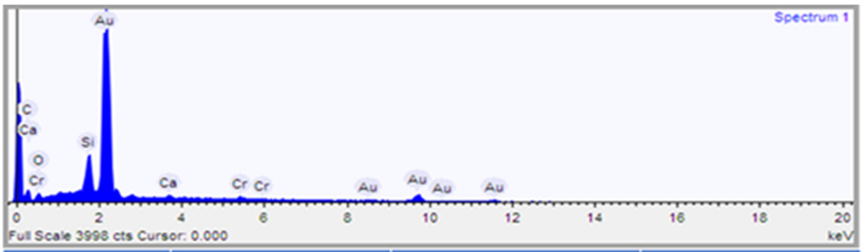

Element	Weight %	Weight % σ	Atomic %
Carbon	5.557	0.724	33.365
Oxygen	6.250	0.391	28.175
Sodium	1.738	0.125	5.451
Chlorine	0.811	0.119	1.650
Gold	85.644	0.767	31.359

(d)

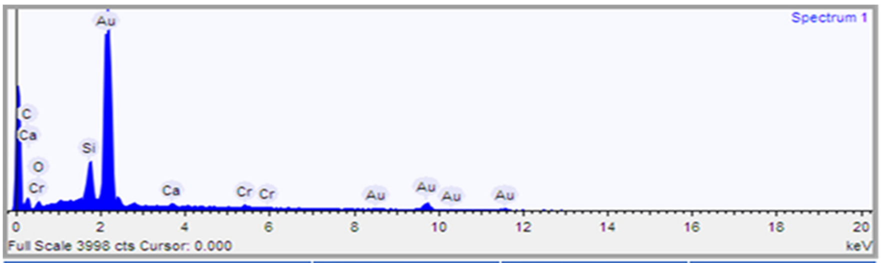

Element	Weight %	Weight % σ	Atomic %
Carbon	10.338	0.602	48.868
Oxygen	5.177	0.494	18.373
Silicon	3.736	0.115	7.553
Calcium	0.810	0.111	1.147
Chromium	1.267	0.167	1.383
Gold	78.671	0.707	22.676

Figure S5. **(a)** EDX of bare Au was done and showed the weight% of gold up to 85.59%; **(b)** weight% up to 86.48% of gold as the deposition of AuNPs on the Au electrode; **(c)** As DTT was added onto the Au/AuNPs electrode, there was a slight change on the surface. Gold weight % was estimated to be 85.64%; **(d)** The deposition of ACR on the Au/AuNPs/DTT fabricated electrode results in a decrease in the weight% of gold up to 78.67%.

6. Sensing of Chips and Coffee Samples Using the Developed Chemosensor

ACR extracted from food samples were added into the electrolyte solutions linearly (10, 20, 30 and 40 μL of acrylamide extracted sample). The measurements were recorded via DPV after adding ACR samples (Figure S6a,b). ACR concentration was quantified using a standard calibration curve and compared with the HPLC method. The concentration was estimated to be 3899 μg/kg (3.89 ppm) of the chip sample. Using HPLC, the ACR concentration was estimated to be 3541 μg/kg (3.541 ppm) of chips samples, comparable to the result obtained by the chemosensor. Similarly, for coffee samples, the estimated concentration of ACR was 1.94 mg/kg (1.94 ppm). In comparison, ACR was estimated with an HPLC value of 1.81 mg/kg (1.81 ppm) for coffee samples. HPLC chromatogram of standard ACR (Figure S7) with a retention time of chips samples was shown in Figure S8. HPLC chromatogram of the coffee sample was determined at 6.35 min, shown in Figure S9. For HPLC, a standard curve using standard ACR solution ranging from 5 to 100 μg/mL was estimated and used for ACR quantification (Figure S10). The equation of the standard curve of HPLC was found to be y = 15672x − 17150 with R^2 = 0.995.

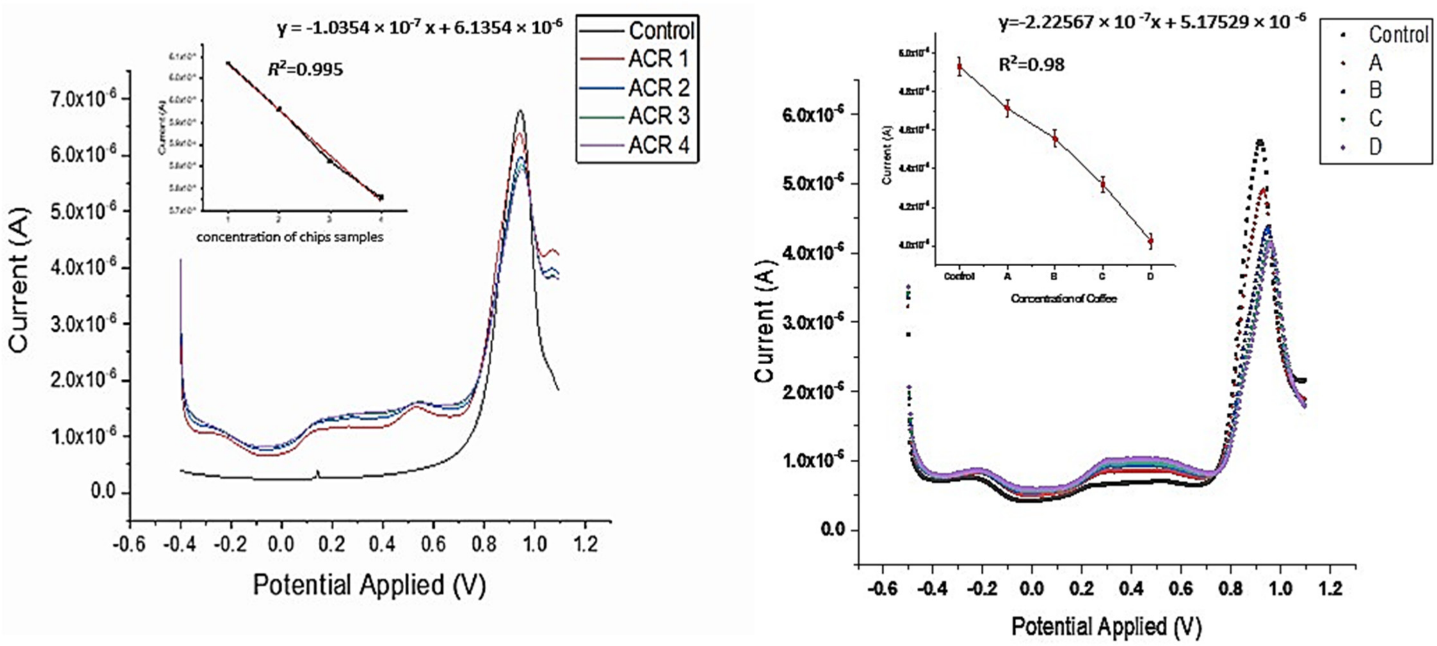

(a) **(b)**

Figure S6. (**a**) DPV curve when chips sample with an unknown concentration was added in the electrolyte solution. (**b**) DPV curve of coffee samples with an unknown concentration differing volume of samples was added in the electrolyte solution.

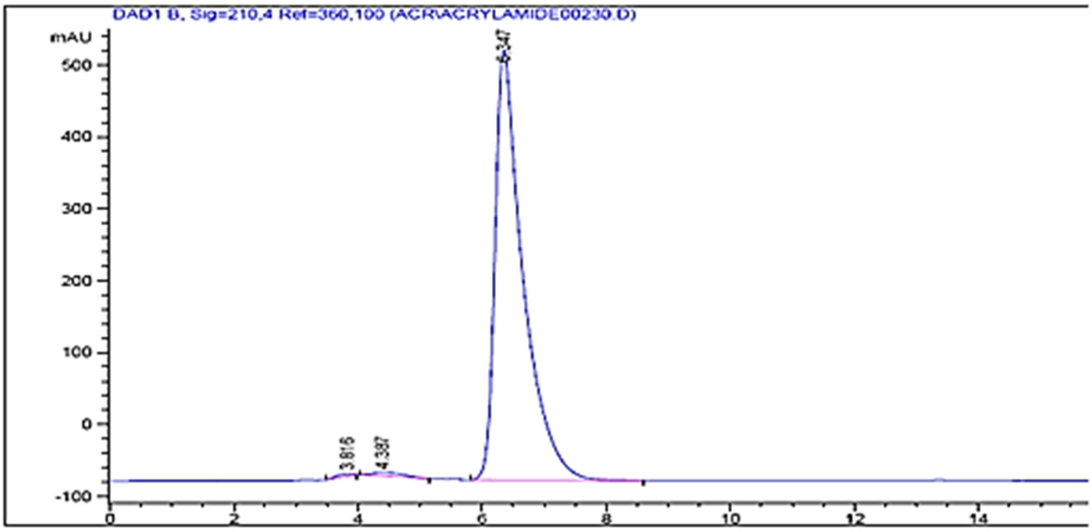

Figure S7. A representative HPLC chromatogram of standard ACR with a retention time of 6.3 min.

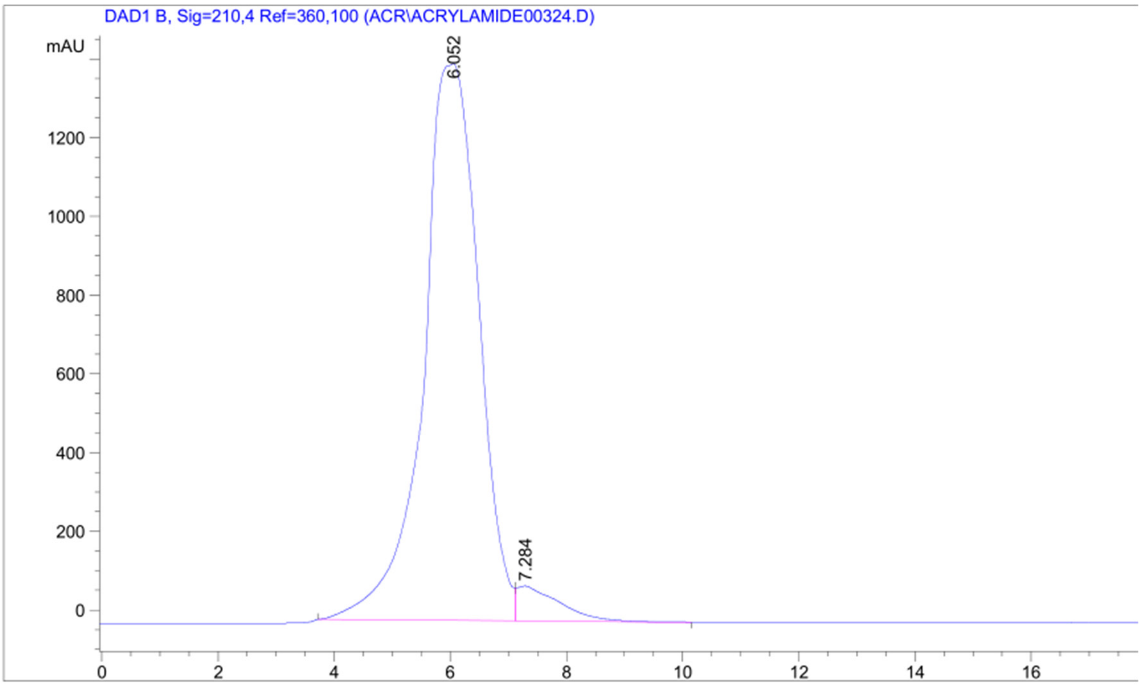

Signal 1: DAD1 B, Sig=210,4 Ref=360,100

Peak #	RetTime [min]	Type	Width [min]	Area [mAU*s]	Height [mAU]	Area %
1	6.052	BV	0.8675	9.38442e4	1415.42578	95.3854
2	7.284	VB	0.6628	4540.06592	88.75031	4.6146
Totals :				9.83843e4	1504.17609	

Figure S8. A representative HPLC chromatogram of the extraction of ACR (Peak Area-93844) from the chip sample with a retention time of 6.0 min.

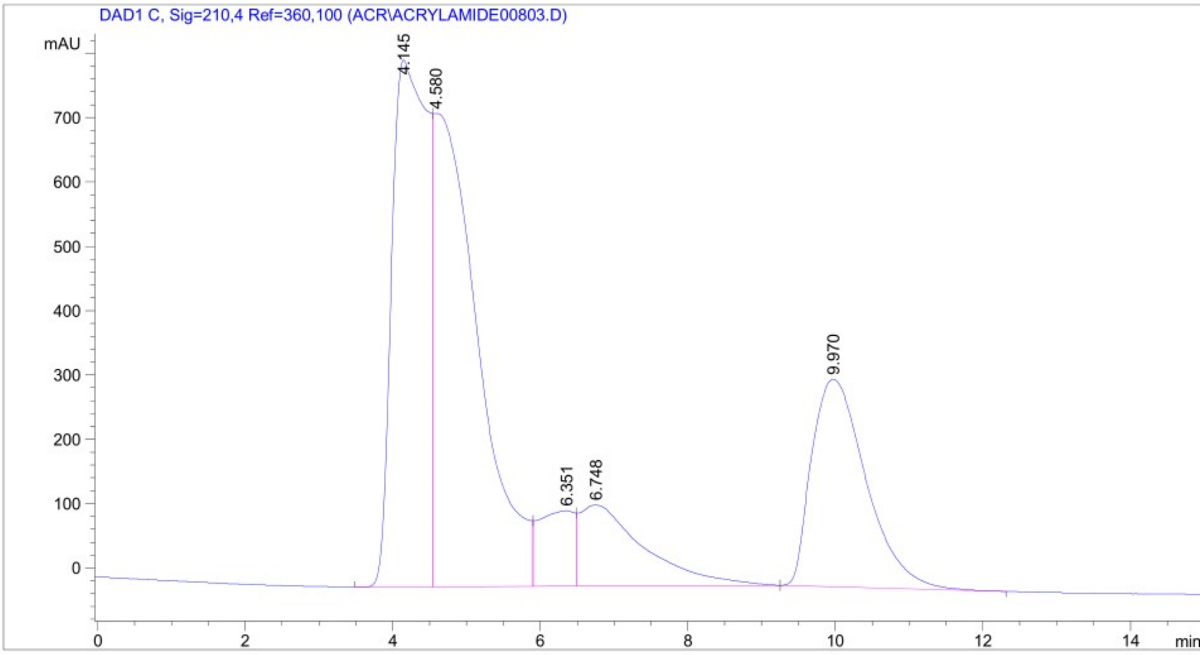

Signal 1: DAD1 C, Sig=210,4 Ref=360,100

Peak #	RetTime [min]	Type	Width [min]	Area [mAU*s]	Height [mAU]	Area %
1	4.145	BV	0.4663	2.78320e4	819.25641	32.1461
2	4.580	VV	0.4963	3.05655e4	736.29688	35.3033
3	6.351	VV	0.4445	3986.92578	117.30493	4.6049
4	6.748	VB	0.7842	7625.01367	125.96016	8.8069
5	9.970	BB	0.8195	1.65702e4	322.47116	19.1387
Totals :				8.65797e4	2121.28954	

Figure S9. HPLC chromatogram of the coffee sample where ACR was observed at a retention time of 6.35 min.

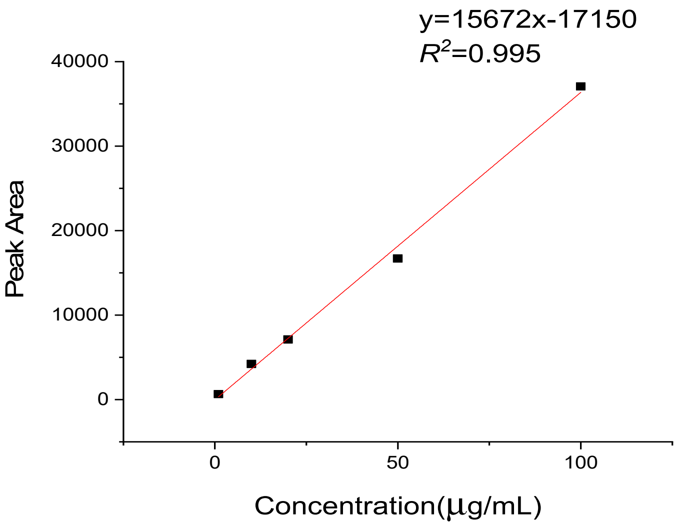

Figure S10. The HPLC calibration curve of the standard ACR with different concentrations (5, 10, 20, 50 and 100 μg/mL).

7. Recoveries of ACR Samples

ACR samples with known concentrations were added to the chips samples and quantified using the chemosensor. The percentage recoveries were determined using the obtained ACR concentration. These results (Figure S10) showed good estimation by the chemosensor without any interference from other endogenous compounds.

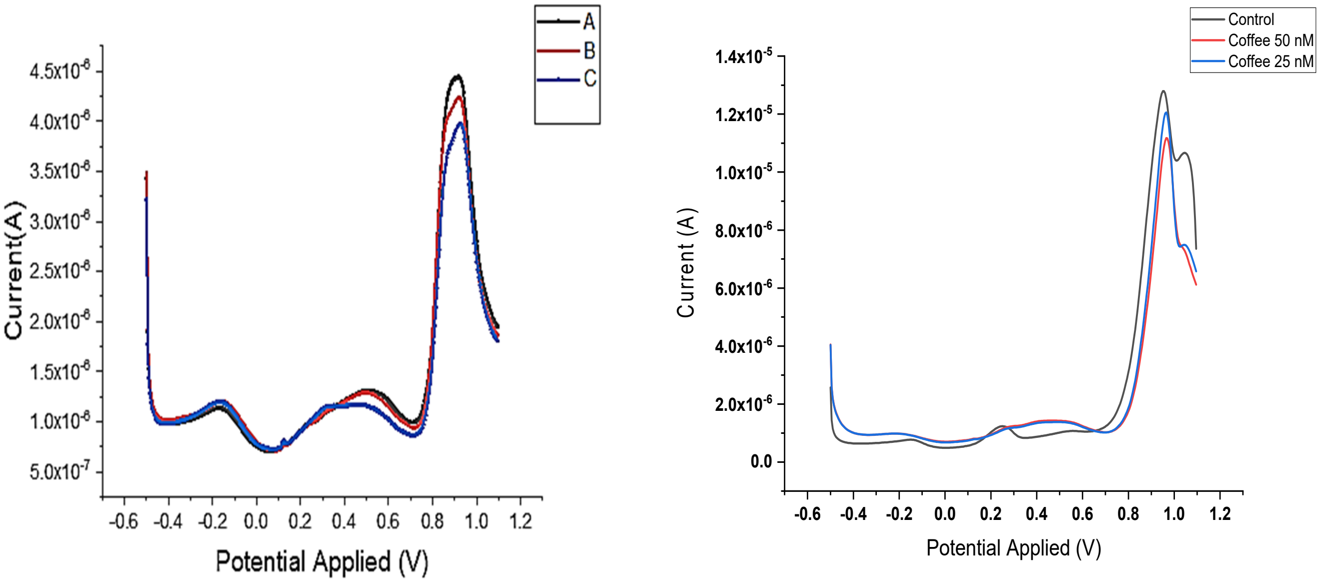

(a) **(b)**

Figure S11. (a) The DPV current peak of ACR was added to chips samples. (A) Peak without the addition of analyte; (B) Addition of 10 nM ACR and (C) 15 nM ACR. **(b)** The DPV current peak of ACR at the addition of ACR with 25 nm and 50 nm concentration.

References

1. Butwong, N.; Khajonklin, J.; Thongbor, A.; Luong, J.H. Electrochemical sensing of histamine using a glassy carbon electrode modified with multiwalled carbon nanotubes decorated with Ag-Ag 2 O nanoparticles. *Microchim. Acta.* **2019**, *186*, 714.
2. Gomez, H.; Riveros, G.; Ramirez, D. Chronoamperometric Cu (II) Analysis at gold ultramicroelectrodes in concentrated sulfuric acid solutions. *Int. J. Electrochem. Sci.* **2017**, *12*, 985-993.
3. Kamat, A.; Huth, A.; Klein, O.; Scholl, S. Chronoamperometric investigations of the electrode–electrolyte interface of a commercial high temperature PEM fuel cell. *Fuel Cells* **2010**, *10*, 983-992.
4. Deletioğlu, D.; Solak, A.O.; Sari, N. Electrochemical behaviour of acrylamide polymers functionalized with some Schiff bases. *Gazi Univ. J. Sci.* **2011**, *24*.
